# Free-living amoebae isolated in the Central African Republic: epidemiological and molecular aspects

**DOI:** 10.11604/pamj.2017.26.57.9021

**Published:** 2017-02-01

**Authors:** Alain Farra, Claudine Bekondi, Vianney Tricou, Jean Robert Mbecko, Antoine Talarmin

**Affiliations:** 1 Institut Pasteur de Bangui, Bangui, Republique Centrafricaine; 2 Institut Pasteur de Guadeloupe, France

**Keywords:** Free-living amoebae, tetramitus, CAR

## Abstract

Among the many species of free-living amoebae infecting humans, only *Naegleria fowleri*, a few species of *Acanthamoeba, Balamuthia mandrillaris* recently *Sappinia diploïdea and Paravahlkampfia Francina* are responsible for human diseases especially deadly encephalitis outside of Acanthamoeba keratitis related. In the Central African Republic (CAR), no studies have previously been conducted about free amoebae and no suspicious cases of encephalitis or amoebic keratitis was reported even though the ecosystem supported the proliferation of these microorganisms. The objective of this study was to identify free-living amoebae present in CAR and to define the molecular characteristic. Bathing sites and cerebrospinal fluid from patients died of bacterial meningitis untagged were explored by culture and PCR and the amplicons were sequenced which allowed to characterize the species found. Only species of the genus *Tetramitus, namely T. Entericus, T. waccamawensis* and *T.sp* similar to those already described in the world and not pathogenic for humans were found in bathing sites, the cerebrospinal fluid meanwhile remained negative. Although no pathogen species such as *Naegleria fowleri* or species of *Acanthamoeba* have been isolated, this study worth pursuing because this investigation was very limited in space because of the insecurity in the country.

## Introduction

Free-living amoebae, unlike parasitic amoebae, complete their entire cycle in nature and do not require a host [1]. Some free-living amphizoic amoebae can, however, accidentally infect humans and cause neurological, ocular and cutaneous infections [2, 3]. The main organisms involved are *Naegleria, Acanthamoeba, Balamuthia*, several amoebae of the genus *Sappinia (S. diploidea, S. pedata)* and a species of the genus *Paravahlkampfia, P. francina*, which was recently incriminated in cases of encephalitis [4–6]. Some of the cases of encephalitis were opportunistic infections in immunodepressed individuals and consisted of granulomatous encephalitis due to *Acanthamoeba* and *Balamuthia*, which evolves chronically and is usually fatal. In contrast, primary amoebic meningoencephalitis due to *Naegleria fowleri* is an acute condition in healthy children and adults, manifesting several days after infection and rapidly evolving to severe disease in the absence of early treatment. In the Central African Republic (CAR), the presence of free-living amoebae has not been studied, and no suspected cases of primary amoebic meningoencephalitis have been reported, although cases have been reported in Nigeria, Zambia and South Africa [7, 8]. The presence in the CAR of hot springs and numerous warm-water lakes with abundant organic matter would indicate that such organisms might exist there also. The objective of this study was to identify and characterize the free-living amoebae that are present and to determine whether they include *N. Fowleri* and try to assess the risk of the population.

## Methods

**Context geographic:** The CAR is a landlocked country in the heart of the African continent in an intertropical zone. It covers an area of 623 000 km^2^ and is bordered on the east by the Republic of Sudan and South Sudan, on the west by Cameroon, on the south by the Democratic Republic of the Congo and the Congo, and on the north by Chad. Its geographical position results in a hot continental climate, with two seasons: a dry season between November and April and a rainy season between May and October. The country has numerous lakes, ponds, rivers and hot springs, in which people swim. We obtained our samples from some of these water bodies, but the insecurity associated with the current military-political situation limited our study to accessible areas near Bangui.

**Figure 1:**
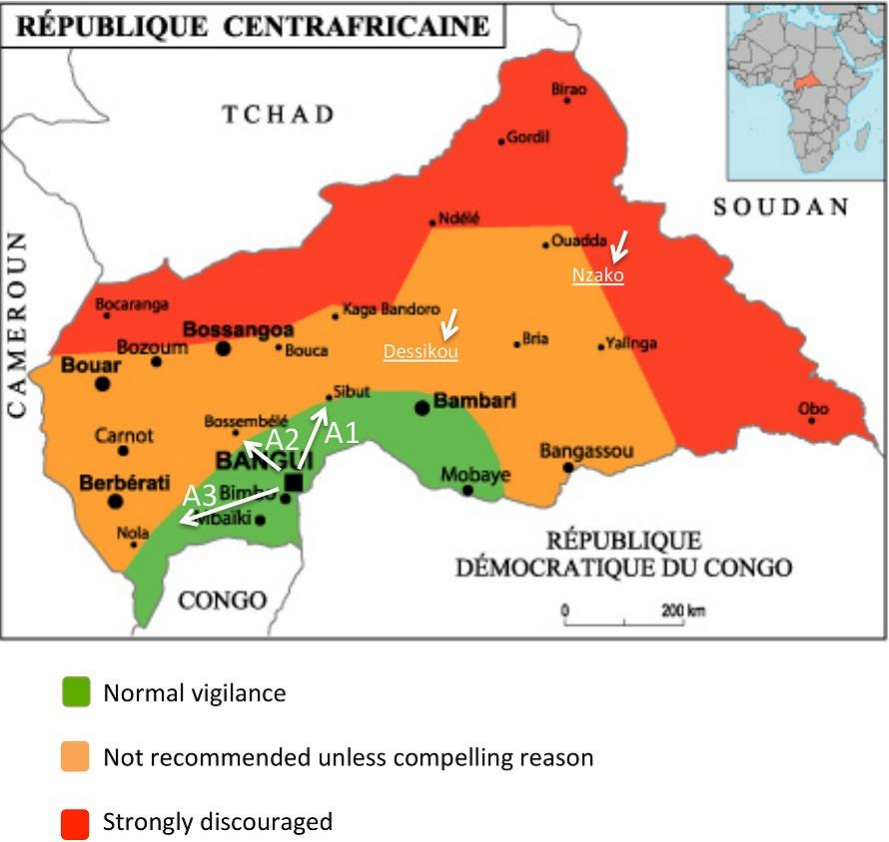
Map of the CAR, showing the directions chosen for sampling and the security situation in the country; This map was made available to all partners in health by the WHO are indicated the different axes (A1: Axis Bangui-Damara-Mbourouba; A2: Axis Bangui-Boali; A3: Axis Bangui-Mbaïki-Mbata) and Dessikou and Nzako cities that could not be investigated

**Sample Collection:** We took samples from 32 sites between 4 April and 23 May 2012 in Bangui and in three directions within a radius of 150 km around the city: Bangui-Damara-Mbourouba, Bangui-Boali and Bangui-Mbaïki-Mbata (Figure 1). The sites contain rivers, lakes, ponds, pools and a few functioning hotel swimming pools in Bangui. The hot springs at Dessikou in Dékoua in the centre of the country and at Nzako near Bria in the east could not be sampled because of security problems. At each site, we noted the temperature and pH of the water and the GPS coordinates. Samples were taken in duplicate for incubation at 37°C and 44 ° C and consisted of 500 ml of water, algae and swab samples from swimming pools. Sediments could be collected only from swimming pools because the bottom of all the other water bodies was mud. The samples were sent to the Institut Pasteur and cultured within 6 h of collection. With the approval of the Ethics Committee, we examined 20 samples of purulent cerebrospinal fluid (CSF) from the biological specimen library at the Institut Pasteur by culture and by PCR to identify any amoebae. These samples were from HIV-negative patients who had died of acute meningoencephalitis for which no bacterial cause had been found by culture or by molecular biology.

**Isolation of Amoebae:** The water samples were filtered with a Millipore^®^ model DOA U152-BN on cellulose nitrate filters with a pore width of 1.2 µm. The filters were cut into eight pieces in a class II microbiological safety cabinet and returned to a non nutrient agar previously flooded with a suspension of *Escherichia coli*, which is used as a nutrient by amoebae. The algae and swab samples were placed directly on the culture medium under the same conditions. The CSF samples were seeded in three spots on the culture medium. All the dishes were then sealed with Parafilm^®^ and incubated at 37°C, at which temperature all free-living amoebae grow, an at 44°C to select for certain species of *N. fowleri*. Reading cultures was made first to the eye and the inverted phase contrast microscope and this every day for at least 5 days. To the naked eye, the colonies of amoebae in the form of an opaque white halo around the deposit to visible white light. They were surrounded with felt on the bottom of the box and then observed under a microscope with a X20 objective which allows to view trophozoïtes and sometimes cysts around the deposits. When a culture was positive, a section of agar containing amoebae was cut out with an öse in the safety cabinet, returned to fresh culture medium and incubated as above.

**DNA Extraction, PCR Amplification and Sequencing:** DNA was extracted from the cultures with a DNA Mini-Kit^®^ (50) (Qiagen) and quantified before PCR with a Qubit 2.0 fluorimeter according to the manufacturer´s instructions. A concentration of 10-20 ng/µl was used for PCR. As morphological identification is difficult, species were identified exclusively by molecular biology with PCR, followed by amplicon sequencing. Three pairs of primers were used respectively for summer generic looking amoebae (JITSFW: 5'-GTCTTCGT AGGTGAACCTGC-3', JITSRV : 5'-CCGCTTACTGATATGCTTAA-3') *Naegleria sp*. (ITSFW : 5'-AACCTGCGTAGGGATCATTT-3', ITSRW: 5'-TTTCCTCCCCTTATTAATAT-3' ) and *N. fowleri*. (NFITSFW: 5'-TGAAAACCTTTTTTCCATTTACA-3', NFITSRV: 5'-AATAAAAGAT TGACCATTTGAAA-3'). The sequencing of the amplicons allowed the diagnosis of species. The amplicons were purified with a QIAquick PCR purification kit (Qiagen) and then sent to GATC Biotech in Germany for sequencing. Analysis with the Blast 2.0 program identified separate species. Sequences were aligned with the Clustal W2 program, and the phylogenetic tree was constructed with MEGA 5.2.2.

## Results

**Figure 2:**
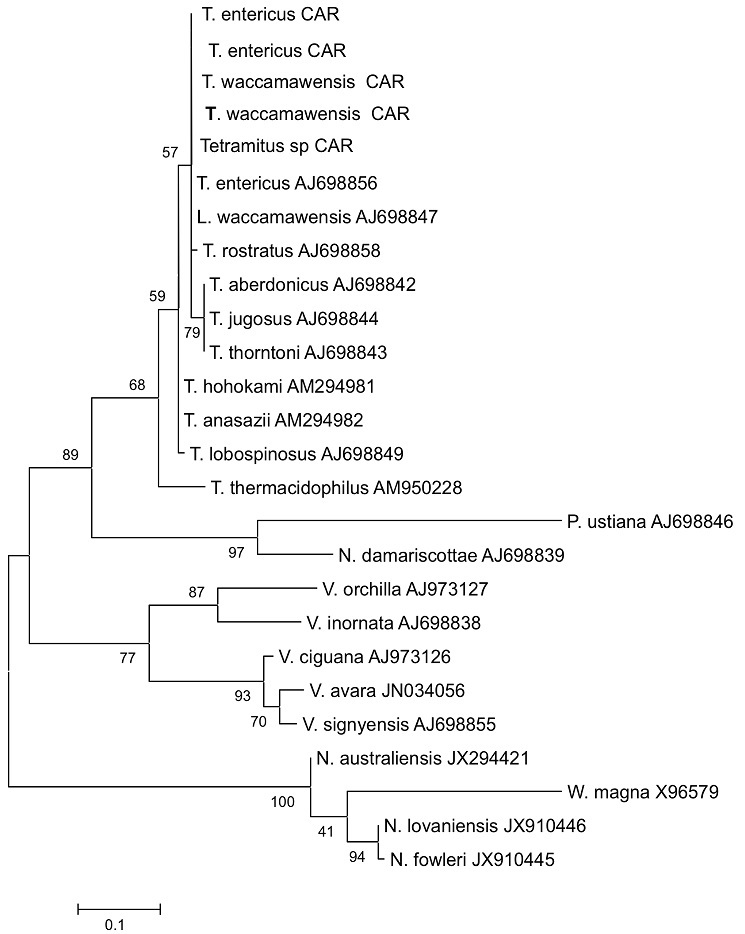
Phylogeny of amoebae found in CAR

The temperature of the water at the sampled sites did not exceed 40°C, and that at 47% of the sites was ≥ 30°C (Table 1,Table 2, Table 3). Samples from only eight of the 32 sites were positive on culture. Five of the cultures were from algae and three from filtered water, but the samples of algae and water were from different sites. Only the cultures incubated at 37°C for 72 h were positive; all those incubated at 44°C were sterile, as were those of CSF. DNA of free-living amoebae was identified by PCR in seven of the eight positive cultures with JITS primers. Naegleria DNA was found in two samples (ITS primers), but *N. fowleri* was not identified (Table 4). Furthermore, no amoebic DNA was found in CSF, and sequencing showed no *Naegleria* species, only the species of *Tetramitus* namely *T. waccamawensis, T. entericius* and *.T. SP.* were identified with the analysis of the sequences (Table 4). The physicochemical characteristics of the various sites did not offer any clues, as the species found were all of the same genus (Table 1,Table 2, Table 3). The phylogenetic study showed that the species found in the CAR were identical to those found in Australia and the USA and were very similar to other *Tetramitus* isolated elsewhere in the world (Figure 2).

**Table 1:** Results of culture and PCR of samples in Bangui

Site	Name	Type	Temperature (°C)	pH	Sample	Results of culture		Results of PCR		
						37°C	44 °C	JITS	ITS	NFITS
Bangui	Ngou Lékpa	Lake	30.3	5.5	Algae	–	–			
Bangui	Ngola	River	29.4	7.1	Algae	–	–			
Bangui	Ngola	River	29.4	7.1	Swab sample	–	–			
Bangui	Ngola F	River	28.2	7.3	Water	–	–			
Bangui	Ngola F	River	28.2		Algae	–	–			
Bangui	Ngola baptême	River	28.5	7.3	Water	–	–			
Bangui	Ngola baptême	River	28.5	7.3	Algae	–	–			
	Landja	Pool	38	6.4	Water	+ on day 3	–	+	–	–
Bangui	Landja	Pool	38	6.4	Swab sample	–	–			
Bangui	Rock Hotel	Swimming pool	31.3	7.5	Water	–	–			
Bangui	Rock Hotel	Swimming pool	31.3	7.5	Swab sample	-	-			
Bangui	Central hotel du Centre	Swimming pool	31,8	7.2	Water	–	–			
Bangui	Central hotel du Centre	Swimming pool	31,8	7.2	Swab sample	–	–			

**Table 2:** Results of culture and PCR of samples on axis 1 and 2

Site	Name	Type	Temperature (°C)	pH	Sample	Results of culture		Results of PCR		
						37°C	44 °C	JITS	ITS	NFITS
	Lac des sorciers	Lake	34	7.7	Water	+ on day 3	–	**+**	–	–
	Kpongorota I	Pool	27.5	7.8	Water	–	–			
	Kpongorota I	Pool	27.5		Algae	–	–			
A1:	Kpongorota II	Pool	32	8.2	Water	–	–			
Axis	Kpongorota II	Pool	32		Algae	+ on day 3	–	**+**	–	–
Bangui-	Soh	Pool	29.5	6.4	Water	–	–			
Damara-	Yangana	Pont	30.4	7.3	Water	–	–			
Mbourouba	Yangana	Pont	30.4	7.3	Swab sample	–	–			
	Gbango	Pont	31.4	7.6	Water	–	–			
	Ngou komba	Pont	29,4	6.8	Water	–	–			
	Boali Pont	Pont	32.1	7.5	Water	–	–			
	Boali Pont	Pont	32.1	7.5	Swab sample	–	–			
A2:	Mpoko	River	27.8	7.3	Water	–	–			
Axis	Mpoko	River	27.8	7.3	Algae	–	–			
Bangui-	Lac des Caïmans	Lake	31.8	7.6	Water	–	–			
Boali	Kodozilo	Pond	27.6	6.8	Water	–	–			
	Kodozilo	Pond	27.6		Algae	–	–			
	Ngou ingo	Pool	28	7.1	Water	–	–			
	Ngou ingo	Pool	28		Algae	–	–			

**Table 3:** Results of culture and PCR of samples on axis 3

Site	Name	Type	Temperature (°C)	pH	Sample	Results of culture		Results of PCR		
						37°C	44 °C	JITS	ITS	NFITS
	Pont Bimon	Lake	25.1	7.5	Algae	+ on day 3	–	**+**	–	–
	Pont Bimon	Lake	25.1	7.5	Water	–	–			
	Mbatama	Lake	35	7.5	Water	–	–			
	Eau Fôret	Lake	28.3	6.9	Water	–	–			
	Eau Fôret	Lake	28.3	6.9	Algae	–	–			
	Eau Sabé	Pond	29	6.6	Algae	+ on day 3	–	+	+	–
	Eau Sabé	Pond	29	6.6	Water	–	–			
A3:	Boyali	Pond	27	7.1	Water	–	–			
Axis	Boyali	Pond	27	7.1	Swab sample	–	–			
Bangui-	Sakoulou	Pond	25	7.5	Water	–	–			
Mbaïki-	Sakoulou	Pond	25	7.5	Swab sample	–	–			
Mbata	Yamboro M	Pond	25,7	7.3	Water	–	–			
	Yamboro F	Pond	27	7.7	Water	+ on day 3	–	–	–	–
	Yamboro F	Pond	27	7.7	Algae	–	–			
	Soke F	Pond	25.4	5	Water	–	–			
	Soke M	Pond	30	5.9	Water	–	–			
	Soke M	Pond	30	5.9	Swab sample	–	–			
	Ngou kossi M	Pond	30.5	6.6	Water	–	–			
	Ngou kossi M	Pond	30.5	6.6	Algae	–	–			
	Ngou kossi F	Pond	28.4	6.5	Water	–	–			
	Ngou kossi F	Pond	28.4	6.5	Algae	+ on day 3	–	**+**	–	–
	Ngou nguere	Pond	27.6	7.3	Algae	+ on day 3	–	**+**	**+**	–
	Ngou nguere	Pond	27.6	7.3	Swab sample	–	–			

*M: male, F: female

**Table 4:** Results of PCR and species identified, by site

PCR positive	JITS	ITS	NFITS	Species identified
Lac des Sorciers	+	-	-	*Tetramitus entericus*
Landja	+	-	-	Not determined
Pont Bimon	+	-	-	*Tetramitus entericus*
Ngou Kossi	+	-	-	*Tetramitus waccamawensis*
Eau Sabé	+	+	-	*Tetramitus waccamawensis*
Ngou Nguere	+	+	-	*Tetramitus* spp.
Pkongorota II	+	-	-	*Tetramitus entericus*

## Discussion

This preliminary search for free-living amoebae in the aquatic environment is the first of its kind in the CAR. It was limited spatially because of the lack of security in the country. The temperature of the water in most of the bodies studied was 25-35°C, and none had a temperature superior at 40°C. No *Naegleria or Acantamoeba* species was isolated, even though these species are ubiquitous and they are probably present in the CAR. The results show that the conditions at certain sites are favourable for the growth of *N. fowleri*, with abundant organic matter and a temperature superior at 30°C; the water at two sites was even superior at 35°C [5, 8, 9]. A case of infection with *N. fowleri* described in Guadeloupe occurred after bathing in water at 27°C, and *N. fowleri* has been found in water at 26.9-34.9°C [10, 11]. Almost all the sites studied were compatible with the presence of this amoeba, which would justify continuation of this study. It will therefore be extended to other sites and particularly hot springs, once the security situation improves. In the cases of primary amoebic meningoencephalitis seen in Madagascar and Guadeloupe, the amoebae were visible in fresh CSF, and PCR of frozen CSF showed the presence of *N. fowleri* [10, 12]. Although our samples were kept at -20°C under good conditions, PCR showed no amoebae. A systematic prospective study of purulent CSF samples with no bacterial cause should be undertaken, with careful direct examination and generic PCR to detect amoebae. The only amoeba species that were isolated belonged to the *Tetramitus* genus in the Vahlkampfidae family. Currently, 17 species have been associated with disease in humans [13]. *T. waccamawensis* was previously classified as *Learamoeba waccamawensis* according to the criteria of Sawyer et al. [14], but a molecular study by Brown and De Jonckheere led to its reclassification in the genus *Tetramitus* on the basis of 98.7% homology with the amoebae of this genus [15]. Except for two species, *T. jugosus* in the marine environment and *T. thermacidophilus* which develops at a pH of 1.2-5 and at temperatures up to 54°C, Tetramitus are usually isolated from freshwater with abundant organic matter, as in the CAR where this amoeba was isolated [16–18]. The two strains identified by sequencing, *T. entericius* and *T. waccamawensis* are similar to the two species already described [13]. The unidentified strain is also similar to these two species; a further study will be conducted to identify this strain.

## Conclusion

As expected, free-living amoebae are present in freshwater in the CAR. They all belong to the *Tetramitus* genus and are not pathogenic to humans. No species of the genera *Naegleria or Acanthamoeba* was isolated, either from the environment or from purulent CSF samples with no bacterial agent. This study should be extended to other sites and particularly to the Dessikou and Nzako hot springs, which could favour the proliferation of *Naegleria*. Better understanding of the ecology and epidemiology of free-living amoebae in the CAR, both in the environment and in HIV-infected patients, is needed, in order to raise awareness about these neglected infections, which are usually fatal, and to put in place prophylactic measures.

### What is known about this topic

- Free-living amoebae and roles in human pathologies;
- Meningoencephalitis caused by *Naegleria fowleri;*
- Amoebic keratitis caused by *acanthamoeba*.

### What this study adds

- Free-living amoebae present in RCA;
- Ecosystem favourable to the presence of *Naegleria fowleri;*
- Possible diagnosis at the Institute Pasteur of Bangui.
